# Aortic Pseudo-dissection

**DOI:** 10.5811/cpcem.2017.9.35658

**Published:** 2017-11-03

**Authors:** Karl Huesgen, Sarah Gul, Candice Norman

**Affiliations:** University of Florida, Department of Emergency Medicine, Gainesville, Florida

## CASE PRESENTATION

A 21-year-old female with a past medical history significant for asthma and oral contraceptive use presented complaining of shortness of breath and wheezing. Symptoms started after contact with a dog. She came to the emergency department (ED) after home albuterol treatments failed to provide relief. Initial vital signs included a blood pressure of 145/49mmHg, pulse rate 127 beats/minute, respirations 32 breaths/minute, temperature 37.1°C (98.8°F), and oxygen saturation of 87% on room air. On auscultation, lung fields demonstrated bilateral wheezing and the expiratory phase was prolonged. She also had retractions and endorsed chest tightness. ED workup included an elevated D-dimer, and subsequent computed tomography (CT) pulmonary angiography indicated ascending aortic dissection instead (Image).

## DIAGNOSIS

### Aortic pseudo-dissection artifact

Emergent preoperative transesophageal echocardiography disproved presence of intimal flap and dissection, so operative repair was aborted. The patient’s tachycardia after multiple beta agonist treatments produced a motion artifact concerning for aortic root dissection. Although CT imaging is highly sensitive and specific for aortic dissections, there is a potential for false-positive ascending dissections (Stanford type A). 1, 2 Such artifacts are frequently seen in the thoracic aorta due to its close proximity to the heart, 3 and tachycardia correlates significantly with motion defects on CT. 4 This problem can be overcome by use of electrocardiography-synchronized (ECG-gated) CT instead. 5, 6 The patient’s asthma exacerbation was treated as an inpatient and she was eventually discharged home. This case illustrates the importance of taking the clinical history along with the patient’s presentation into account when making a diagnosis.

**CPC-EM Capsule**

What do we already know about this clinical entity?

An aortic dissection occurs when blood enters the medial layer of the aortic wall through a tear in the intima. An aortic pseudo-dissection on the other hand occurs due to aortic pulsation motion artifact on imaging.

What is the major impact of the image(s?)

An inaccurate diagnosis of an aortic dissection might result in a patient undergoing unnecessary emergent surgery.

How might this improve emergency medicine practice?

There is a risk for false-positive computed tomography (CT) results with ascending dissections. Using electrocardiography-gated CT is useful and may prevent unnecessary surgery. It is also important to take the patient’s history and presentation into account and not rely on imaging alone when making a final diagnosis.

## Figures and Tables

**Image:**
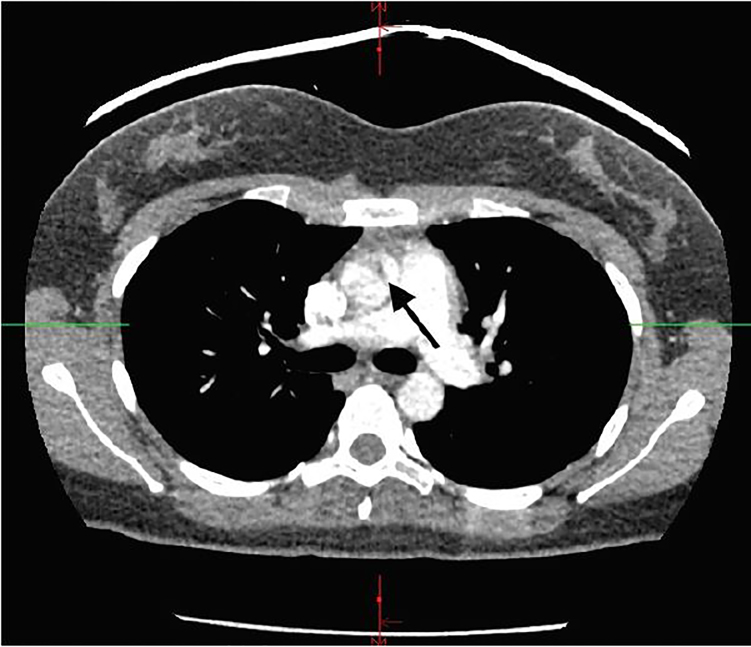
Motion artifact suggesting luminal flap of aortic dissection (arrow).
